# Sonographic Appearance of Lacrimal Glands in Sjögren’s Syndrome at High Risk for Lymphoma Development

**DOI:** 10.7759/cureus.50047

**Published:** 2023-12-06

**Authors:** Ivan Giovannini, Enrico Pegolo, Simone Longhino, Luca Quartuccio, Alen Zabotti

**Affiliations:** 1 Rheumatology, Azienda Sanitaria Universitaria Friuli Centrale, Udine, ITA; 2 Institute of Anatomic Pathology, Azienda Sanitaria Universitaria Friuli Centrale, Udine, ITA

**Keywords:** lacrimal gland ultrasonography, cryoglobulinaemia, parotid gland biopsy, lymphoma, parotid gland swelling, salivary gland ultrasonography, primary sjogren syndrome (pss)

## Abstract

Primary Sjögren’s syndrome (pSS) is an autoimmune systemic disease characterized by the destruction of exocrine glands, mainly salivary and lacrimal glands. The diagnosis is generally made upon objective tests aimed at assessing salivary and lacrimal glandular function, autoantibody assays, and the results of labial salivary gland biopsies. A major salivary gland biopsy is usually reserved to assess lymphoproliferative complications. Recently, the sonographic evaluation of the major salivary glands has gained a crucial role in assessing the glandular parenchyma and early detecting abnormalities, while the role of ultrasonography in the assessment of lacrimal glands is still secondary. Our case report is about a male patient who presented parotid gland swelling and purpuric lesions, with preserved salivary and lacrimal glandular function. Considering the presence of risk factors associated with lymphoproliferative development and the peculiar characteristics detected by salivary and lacrimal gland ultrasonography, we performed a parotid gland biopsy, confirming Sjögren’s syndrome. Our case demonstrates that lacrimal gland ultrasonography could be implemented, along with major salivary gland ultrasonography, as a routine procedure in evaluating patients with suspected or definite diagnoses of pSS.

## Introduction

Primary Sjögren’s syndrome (pSS) is a systemic autoimmune disease that primarily targets the exocrine glands, mainly salivary and lacrimal glands. It is characterized by immune-mediated destruction of exocrine glands, B-cell hyperactivation, and excessive infiltration of inflammatory cells, thus leading to the progressive loss of glandular function and an increased risk of lymphoproliferative disorder [1]. The incidence rate for pSS is 6.9 per 100,000 person-years, and the overall prevalence is about 60 cases per 100,000 inhabitants [2].

The pSS diagnosis is generally made upon objective tests aimed to assess salivary and lacrimal glandular function, autoantibody assays, and eventually the results of labial salivary gland biopsies [3,4]. A major salivary gland biopsy is usually reserved to evaluate lymphoproliferative complications [5,6] since the diagnosis of lymphoma needs to be pathologically proven. In recent years, sonographic evaluation of the parotid and submandibular glands has attracted increased interest, allowing the assessment of the glandular parenchyma and the detection of salivary gland abnormalities [7,8]. However, the role of this technique in assessing lacrimal glands is still secondary [7,9,10].

Here, we report a case of a 48-year-old man who presented multiple factors linked to lymphoma development in Sjögren’s syndrome.

## Case presentation

We report the case of a 48-year-old man who presented at our rheumatology department with a new onset of symmetric purpuric lesions in lower limbs and a long history of fever, myalgia, and arthralgia. In the previous two years, he reported recurrent episodes of unilateral parotid swelling, occurring alternatively on both parotid glands. He underwent elsewhere minor salivary gland biopsy, which concluded non-specific chronic sialadenitis, and he was diagnosed with acute bacterial sialadenitis.

The patient was referred to our department due to the recurrence of parotid swelling and purpuric lesions in the lower limbs. Salivary and lacrimal glandular function was preserved. At the laboratory examination, we detected rheumatoid factor positivity, type II cryoglobulinemia, mild polyclonal hypergammaglobulinemia, C3/C4 hypocomplementemia, and high titer ANA positivity (1:1280 speckled) with anti-Ro52/60 and anti-La specificity. Hepatitis C serology, antiphospholipid antibodies, anti-DNA antibodies, and anti-citrullinated peptides antibodies were negative.

**Figure 1:**
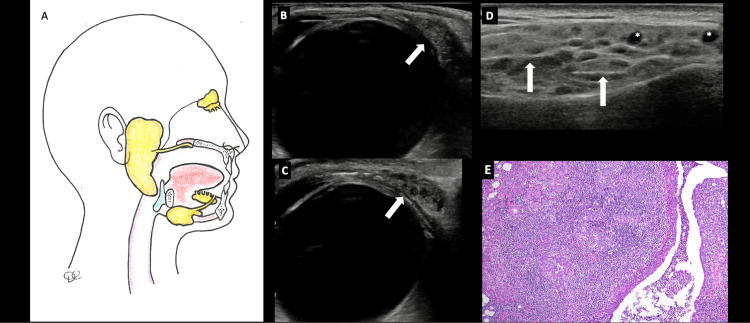
(A) Anatomical representation of the salivary and lacrimal glands affected by primary Sjögren’s syndrome (pSS), leading to oral and ocular dryness and to glandular swelling. Original image created by the author. (B) Ultrasound image of a healthy subject’s lacrimal gland (arrow). Image obtained with Samsung RS85, linear probe LM4-18B. (C) Ultrasound image of the patient’s lacrimal gland (arrow) with severe parenchymal inhomogeneity and increased volume. Image obtained with Samsung RS85, linear probe LM4-18B. (D) Ultrasound image of the patient’s parotid gland with severe parenchymal inhomogeneity, with hypoechoic lesions (*) and hyperechoic bands (arrows). Image obtained with Samsung RS85, linear probe LM4-15B. (E) Histopathological image of the parotid gland showing lymphoepithelial sialadenitis (LESA) (H&E, 100x magnification).

Major salivary gland ultrasound (SGUS) showed parenchymal inhomogeneity of the major salivary gland (outcome measures in rheumatology - OMERACT score 3 for parotid and submandibular glands) (Figure 1D). The lacrimal gland ultrasound (LGUS) showed bilateral enlarged lacrimal glands (Figure 1A) with severe parenchymal inhomogeneity due to the presence of a hypoechoic lesion and increased vascularization (Figures 1B, 1C), resembling the abnormalities found in the major salivary glands.

Considering the presence of risk factors for lymphoproliferative development, we performed a parotid gland biopsy to rule out a concomitant lymphoma. The pathological examination highlighted various lymphoid foci, germinal centers, lymphoepithelial lesions, and a focus score of 3.6, leading to the histopathological diagnosis of lymphoepithelial sialadenitis (LESA) (Figure 1E).

The final diagnosis of pSS with associated cryoglobulinemic vasculitis was performed. The patient was treated with rituximab, showing a marked improvement, especially in the purpuric lesions. Subsequent ultrasound examinations of the lacrimal and parotid glands showed no noteworthy variations at the one-year mark following treatment so far.

## Discussion

In recent years, SGUS is playing an increasingly important role in pSS. Indeed, if Schirmer’s test and sialometry are useful tools to assess lacrimal and salivary gland function, they cannot evaluate parenchymal abnormalities, which are detectable only by imaging techniques, such as ultrasonography and magnetic resonance imaging [11]. Many authors, such as Jousse-Joulin et al. [12], Ramsubeik et al. [13], and Lorenzon et al. [14], have published about the role of SGUS in pSS. However, they focused mainly on the assessment of major salivary glands, with lacrimal glands receiving less attention.

Furthermore, in clinical practice, physicians tend to evaluate lacrimal glands only partially through objective tests, such as Schirmer and breakup time (BUT), which can be impaired also in conditions such as age or drug-related dry eye syndromes [15,16].

Our case report highlights the glandular involvement of both salivary and lacrimal glands in the course of pSS. Owing to the technical progress in developing more accurate ultrasound machines and higher frequency transducers, the sonographic assessment of lacrimal glands may be performed routinely, along with SGUS. Indeed, until 20 years ago, Giovagnorio et al. reported that up to 60% of pSS patients had lacrimal glands impossible to detect by LGUS [17]. Today, with new ultrasound machines, we can assess various Sjogren-related pathological features [10]. In fact, De Lucia et al. reported that LGUS has good-excellent inter- and intra-rater reliability scores in pSS and identified parenchymal inhomogeneity and fibrous appearance as key features able to distinguish between healthy and Sjogren’s lacrimal glands [18]. LGUS might assess and differentiate inflammatory from damage-related lesions and distinguish patients with pSS from those with idiopathic sicca syndrome [10]. Furthermore, LGUS may recognize anatomic alterations such as lacrimal gland masses and possible manifestations of lacrimal lymphoma.

## Conclusions

In conclusion, LGUS could be implemented along with SGUS as a routine procedure in evaluating patients with suspected or definite diagnoses of pSS. Associated with clinical features, LGUS and SGUS may provide the basis of a new assessment and diagnostic approach to suspected pSS patients.
